# Realtime user ratings as a strategy for combatting misinformation: an experimental study

**DOI:** 10.1038/s41598-023-28597-x

**Published:** 2023-01-28

**Authors:** Jonas Stein, Vincenz Frey, Arnout van de Rijt

**Affiliations:** 1 Department of Sociology , University of Groningen, Groningen, The Netherlands; 2 Department of Sociology, European University Institute, Fiesole, Italy; 3 Department of Sociology, Utrecht University, Utrecht, The Netherlands

**Keywords:** Human behaviour, Computational models, Information technology

## Abstract

Because fact-checking takes time, verdicts are usually reached after a message has gone viral and interventions can have only limited effect. A new approach recently proposed in scholarship and piloted on online platforms is to harness the wisdom of the crowd by enabling recipients of an online message to attach veracity assessments to it. The intention is to allow poor initial crowd reception to temper belief in and further spread of misinformation. We study this approach by letting 4000 subjects in 80 experimental bipartisan communities sequentially rate the veracity of informational messages. We find that in well-mixed communities, the public display of earlier veracity ratings indeed enhances the correct classification of true and false messages by subsequent users. However, crowd intelligence backfires when false information is sequentially rated in ideologically segregated communities. This happens because early raters’ ideological bias, which is aligned with a message, influences later raters’ assessments away from the truth. These results suggest that network segregation poses an important problem for community misinformation detection systems that must be accounted for in the design of such systems.

## Introduction

Twenty percent of the time users spend consuming news on the four largest social media sites, they are looking at content linking to one of 98 websites that researchers, professional fact-checkers and journalists agree produce fake, deceptive, low-quality or hyperpartisan news^1^. This figure excludes misinformation that is produced less systematically, e.g. by news sites that only occasionally err or deceive, or by users themselves. So how can the propagation of such information on social media be mitigated? Extant approaches include algorithmically aided misinformation detection^2–4^, professional fact-checking with subsequent flagging or retraction^5,6^, and crowdsourced veracity assessments^7–9^. An issue with these approaches is speed. Professional and crowd-based fact-checking takes time and many algorithms cannot act instantly as they must first gather behavioral data. Until a verdict is reached so that a piece of false information can be flagged or retracted, potentially false information can spread unchecked.

An emergent approach in the scientific literature on misinformation^8,10–12^ as well as in practice is to harness the wisdom of the crowd by enabling recipients of an online message on a social media platform to attach veracity assessments to it. This may allow poor initial crowd reception to temper belief in and further spread of misinformation. For example, on Twitter’s Birdwatch users can write notes and attach these to a Tweet, explaining why they believe it is or is not misleading. Other users can rate these notes or write additional notes in response.

The main challenge this approach must overcome is to somehow function in online environments where truth seeking is not the dominant driver of behavior, but rather personal convictions, e.g. of a political or ideological nature. Previous studies on misinformation have shown that sharing decisions regarding messages with a clear political leaning are primarily guided by users’ ideological congruence with the message and only little by perceived veracity^13–15^. Nonetheless, previous work on the wisdom of the crowd shows that also when individuals have strong individual biases of an ideological or other nature, as long as the average individual’s assessment is better than random, the aggregation of judgments produces an accurate collective assessment. This work assumes that individuals in a crowd cast independent votes^16,17^, and it suggests that while individual judgements may not be very accurate, their average often closely approximates the truth^7,18–20^. Recent experimental studies further show that when individuals do not make true-or-false decisions independently but are influenced by the decisions of those who came before them – as they would be on social media – individuals’ accuracy further improves^21–25^. This is so because as long as the average decision-maker is more accurate than random, prior decisions of others will tend to nudge decision-makers towards the truth. If a developing rating starts off with the majority of decisions being correct, this will influence subsequent users towards making the correct decision and thereby further improve the rating. It can of course happen that the same social influence dynamics facilitate the spread of a false belief, namely when the initial decisions are incorrect. Subsequent users may then be influenced to make incorrect decisions themselves, further solidifying the incorrect rating.

In previous studies on social influence and the wisdom of crowds^19,22,26–28^, only chance could generate a large early majority favoring the wrong veracity verdict, because in these studies either all subjects first cast an independent vote and then could revise based on the first round results, or the order according to which subjects made decisions was random. In online social networks contexts, however, the order according to which subjects would cast veracity verdicts occurs along the path through which information disseminates. And herein lies the problem: Such an order is far from random. Online social networks, like most social networks, are homophilous^29,30^, comprising communities of predominantly like-minded peers^31–33^. The level of segregation rarely reaches the extremity implied by the terms ‘echo chambers’ or ‘filter bubbles’ but is nonetheless substantial^34–37^. Different groups of online users have different levels of ability to identify misinformation^4,14,38,39^, and this ability correlates with political biases^2^ and demographic characteristics^40^. Misinformation is often politically or ideologically charged, or intentionally designed to mislead only a specific part of the population^15,41^, and it usually appears among and targets those clusters of users who are most susceptible to it. Hence it would then appear that ratings would have to be able to cope with misinformation initially being rated in communities of individuals who all tend to have the same biases and likely believe the misinformation or in bad faith misclassify it as true.

**Figure 1:**
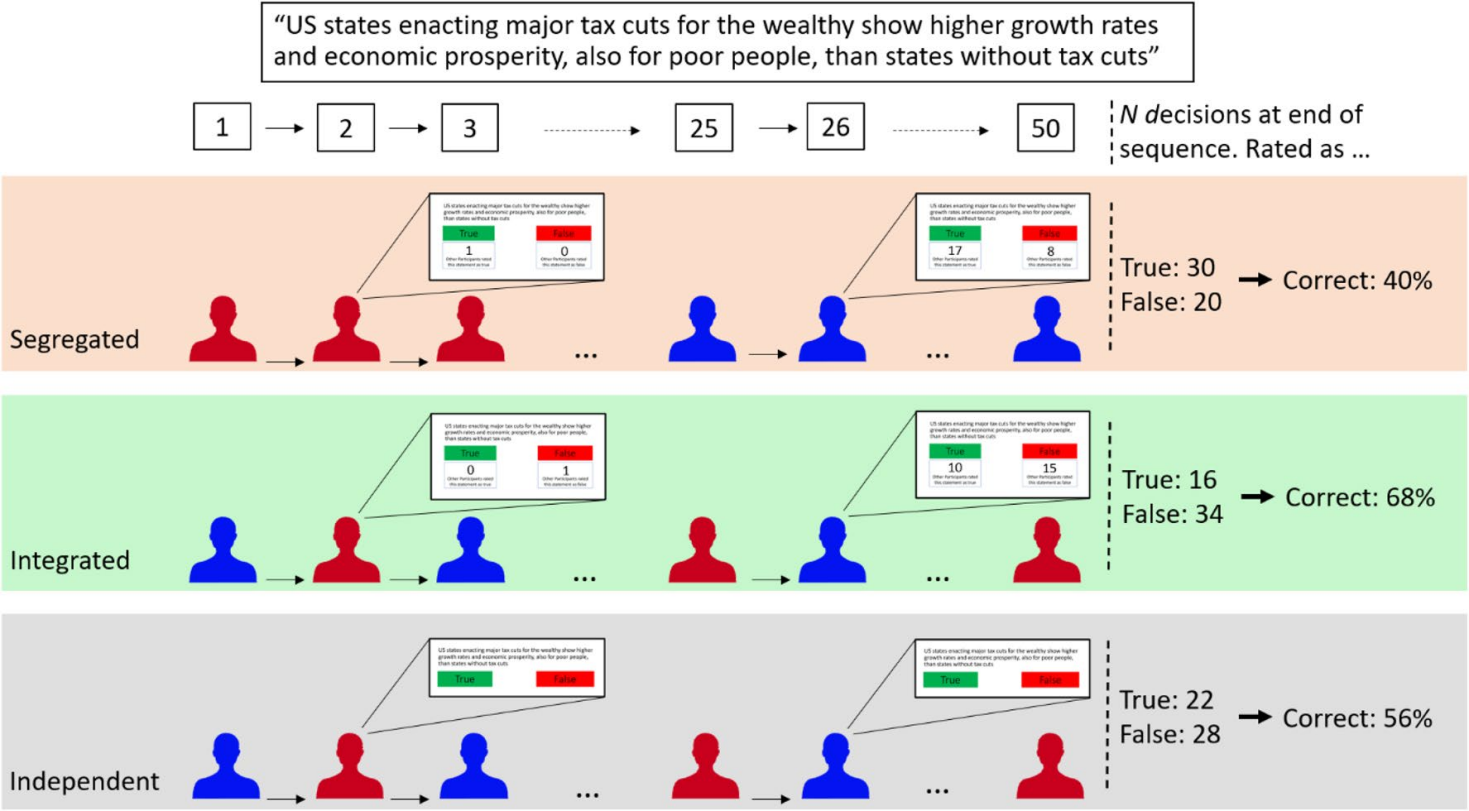
Study setup. Subjects were randomly assigned to ideologically segregated, ideologically integrated, or independent groups and instructed to rate 20 informational messages as true or false. Subjects rated messages sequentially. In segregated and integrated groups, subjects were presented a count of previous group members’ rating decisions. Subjects in independent groups made rating decisions without being exposed to previous subjects’ decisions. Red (blue) icons represent self-identified conservatives (liberals). The example message in the figure is false and has a conservative leaning. For each rating group, we measured how many correct and incorrect rating decisions were made (right side of the figure, data taken from exemplary groups observed in the experiment). We collected data from 80 rating groups with 50 subjects each, amounting to a total of 80,000 rating decisions.

Our study explores real-time user ratings under such circumstances in a large-scale experiment with 2000 liberal and 2000 conservative subjects in 80 bipartisan groups (Fig. 1). We implement two scenarios in which ratings are broadcast immediately after launch: First, a scenario mimicking the development of a real-time rating in an ideologically *integrated* network marked by many cross-partisan ties and no clustering according to ideology. Second, we implement a scenario representing the typical rating sequence in an ideologically *segregated* network, with individuals whose ideology is aligned with the content of a message rating the message first and more critical individuals rating the message later. These scenarios represent ideal-types and maximize our treatment as extreme cases of a continuum along which more and less segregated real-world online communities are positioned^30,32^. We further compare these scenarios with a control condition resembling the setup in which crowd-based ratings have been studied previously^7,9^: namely, a scenario in which subjects rate messages independently and without information about the rating decisions of others.

## Theoretical model and expectations

The simulation model we introduce in this section predicts that if groups are ideologically integrated, broadcasting the rating will trigger a positive feedback loop that improves individuals’ capacity to differentiate between true and false messages. This happens despite strong ideological bias for or against such messages. Similarly, if *true* information is rated in segregated groups, early ratings from individuals with an ideological bias in favor of the true message foster the development of a correct rating. However, broadcasting the rating backfires and reduces correct identification when *false* information is first rated exclusively by ideologically friendly users and only later by ideologically opposed individuals.

In our model, individuals 1 ≤ *i* ≤ *n* make a binary rating decision *C*_*i*_ with regards to an informational message *m* with veracity *v* = 1 if the message is true, or *v* =  − 1 if the message is false. Ratings are made sequentially. Individuals’ propensity to make a correct rating decision $Prob\left({C}_{i}=1\right)$ is given by the following logistic function:

1 $$Prob\left({C}_{i}=1\right)= {\left(1+ \frac{{d}_{i}}{{1-d}_{i} } {e}^{ -s \times { r}_{i}}\right)}^{-1}.$$

The propensity to correctly classify is negatively impacted by how difficult it is to correctly classify a certain message. This difficulty, *d*_*i*_, is the probability of incorrectly classifying a message when this is done independently, in the absence of information from others (0 ≤ *d*_*i*_ ≤ 1). *d*_*i*_ takes on the value of *d*_*align*_ for ideologically aligned individuals and *d*_*mis*_ for misaligned individuals. The difficulty terms *d*_*align*_ and *d*_*mis*_ capture ideological bias stemming from cognitive mechanisms such as motivated reasoning^42,43^ and confirmation bias^44^: It is more difficult for aligned individuals to identify a false (aligned) message as false, but less difficult for misaligned individuals to identify a false (misaligned) message as false $(v=- 1\to {d}_{align}> {d}_{mis})$. Likewise, cognitive bias makes it less difficult for aligned individuals to find true information true, but more difficult for misaligned individuals $(v=1\to {d}_{align}< {d}_{mis})$. Formally, ${d}_{align}= \overline{d }-(b\times v)/2$ and ${d}_{mis}= \overline{d }+(b\times v)/2$, where $\overline{d }$ denotes the average level of difficulty in the population. As we use an equal number of aligned and misaligned individuals in each simulation as well as in the experiment we report on later, $\overline{d }= \frac{({d}_{align} + {d}_{mis})}{2}$*.*The term *b* captures to what extent a message activates bias in individuals (0 ≤ *b* ≤ 1) and corresponds to the absolute difference in difficulty between aligned and unaligned individuals: *b* =| *d*_*align*_ – *d*_*mis*_ |. Individual *i*’s propensity to correctly rate a message further depends on the previous classification decisions of others through the rating *r*_*i*_, which is the average of previous decisions (Eq. (2)).

2 $${r}_{i, i > 1}=\frac{{\sum }_{j< i}{c}_{j}}{i - 1 },$$

*r*_*i*_ ranges from − 1 (= all prior user classifications were incorrect) to + 1 (= all prior classifications were correct). For the first individual, *i* = 1, *r*_*i*_ equals 0. *s* denotes the degree to which individuals are influenced by rating *r*_i_. We assume positive susceptibility to the rating (*s* > 0), which implies that $Prob\left({C}_{i}=1\right)$ monotonically increases with *r*_*i*_, and we assume that everyone is equally susceptible to social influence.

We derive hypotheses through simulation of this model. Each simulation run starts with the first individual *i* = 1, making a first rating decision with *Prob*(*C*_*i*_ = 1) = 1 $-$ *d*_*i*_ in the absence of prior ratings. The decision of *i* factors into the rating signal of the next individual *i* + 1, *r*_*i*+1_, influencing *i* + 1’s rating decision. The simulation stops after *i* = *N* has made their decision. We match population sizes of our simulations with those in the experiment (*N* = 50). Similar results are obtained for smaller and larger populations. Simulation runs are executed 10,000 times for each parameter combination of interest. The dependent variable is the fraction of correct rating decisions out of all rating decisions, computed as an average of fractions over many simulation runs. We choose a target value that reflects average performance rather than a group decision because real-time ratings do not intend to reflect a final verdict (such as a majority vote) but aim to improve raters’ information detection capabilities.

We investigate the interplay of rating order, message veracity and cognitive biases in two real-time rating scenarios in which ratings are broadcast immediately: In the *segregated scenario*, a message originates and spreads in the aligned cluster so that aligned individuals sequentially rate first. The message then reaches the misaligned cluster and misaligned individuals rate it until everyone in the population has made their decision. In the *integrated scenario*, aligned and misaligned individuals alternate in making ratings. These scenarios are compared with an *independence scenario* in which choice order is alternating as well but in which the rating is not broadcast so that individuals make choices without knowledge of others’ ratings (i.e. *s* = 0 implying $Prob\left({C}_{i}=1\right)=1-{d}_{i}$ ∀ *i*).

**Figure 2:**
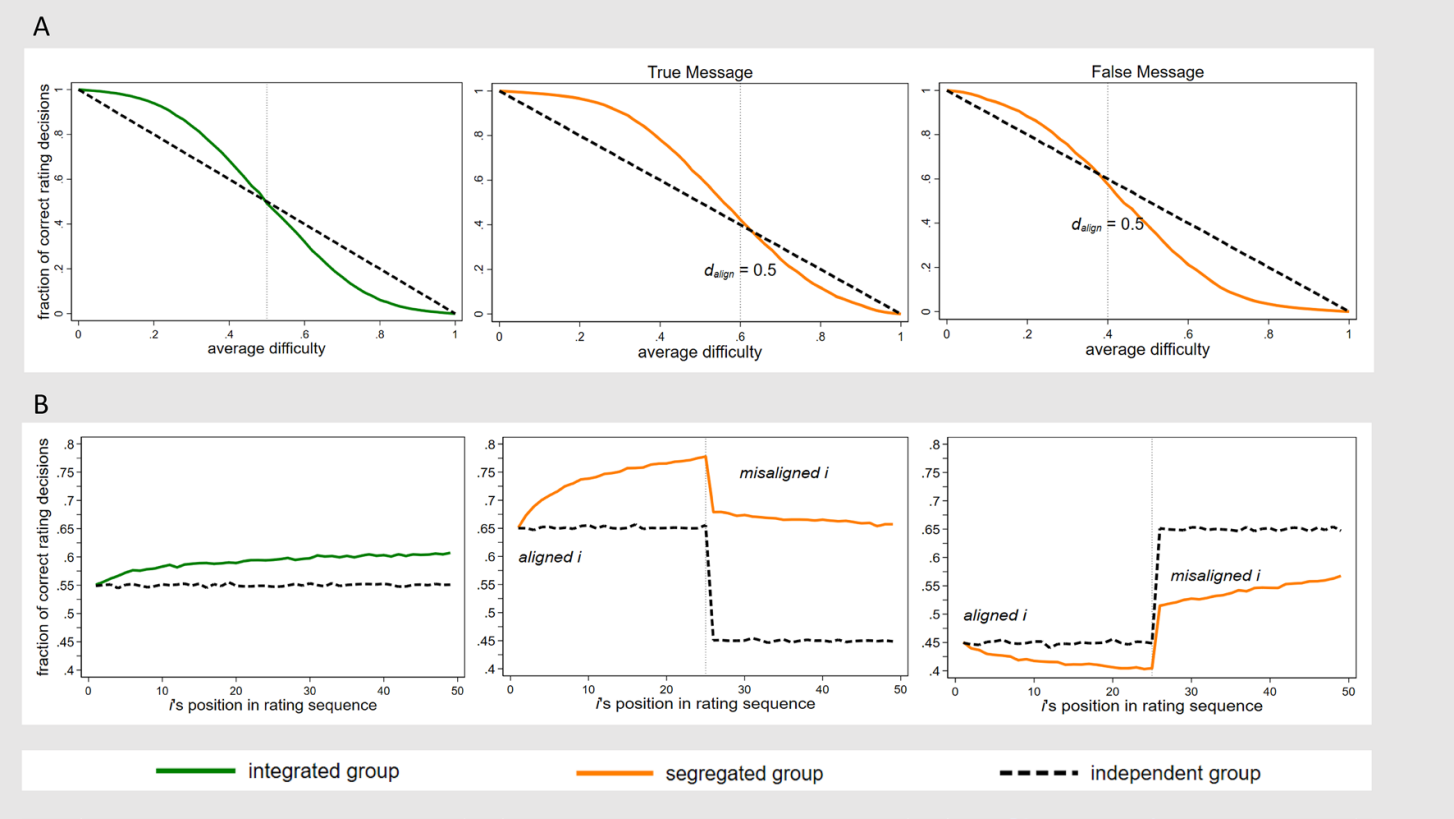
Theoretical expectations. We simulate sequences of rating decisions in 30,000 groups of bipartisan agents (25 ideologically aligned and 25 misaligned agents per group). The fraction of correct rating decisions is shown (**A**) as a function of difficulty and (**B**) as a function of an agents’ position in the rating sequence. In integrated groups, agents classify messages more often correctly than those in independent groups when average difficulty $\overline{d }$ < 0.5, irrespective of message veracity. In segregated groups, agents classify messages more often correctly than those in independent groups if true messages are being rated but classify messages less often correctly if false messages are rated. Parameters used in all panels: | *d*_*align*_—*d*_*mis*_ |= *b* = 0.2, *v* = [− 1; 1], *s* = [0; 2.35]. Panel B: $\overline{d }$ = 0.45.

In the independence scenario, the fraction of correct rating decisions equals the inverse of the average level of difficulty in the population, i.e. 1 – $\overline{d }$. In the integrated scenario, it is to be expected that more individuals will make correct rating decisions than in the independence scenario if $\overline{d }$ < 0.5 and fewer if $\overline{d }$ > 0.5 (Fig. 2A, left). Namely, if $\overline{d }$ < 0.5, the first individual is more likely to make a correct rather than a false rating decision. If the first individual makes a correct rating decision, they influence the following individual to make a correct decision themselves, which enhances the accuracy of the rating for the next individual, and so forth. A real-time rating triggers a positive feedback loop for $\overline{d }$ < 0.5, where each subsequent *ith* rating has a higher probability to be correct than the previous one (compare Fig. 2B, left). Individual biases cancel each other out in the alternating ratings of aligned and misaligned individuals. These theoretical expectations hold for true as well as false messages equally since we assume no systematic differences in information difficulty between true and false information. A negative feedback loop, or ‘backfiring’, on the opposite, is expected to be triggered for $\overline{d }$ > 0.5 since individuals are more likely to make incorrect rather than correct decisions. We accordingly formulate Hypothesis 1:

*H1: When it is not too difficult to classify a message correctly (*$\overline{d }$ < *0.5), then individuals in integrated groups (with information about previous rating choices) classify true and false messages more often correctly than individuals in independent groups (without information about previous rating choices).*

In the segregated scenario, aligned individuals give ratings first. Since they align with the standpoint of a given message, they are more likely to correctly identify a true message as true. On the other hand, compared to misaligned individuals, they have greater difficulty identifying a false message as false. Since aligned individuals are the ones to rate first, their decisions will determine the early accuracy of the rating signal and influence later raters. If messages are true and difficulty among aligned individuals *d*_*align*_ is below 0.5, the rating is likely to enter a positive feedback loop. Later misaligned raters – although less likely to make correct rating decisions due to their bias ‒ will make a correct decision more often than those raters without exposure to a rating signal (Fig. 2A, center). If messages are false and *d*_*align*_ is instead above 0.5, early raters are likely to make incorrect rating decisions and the rating is expected to backfire, resulting in a lower fraction of correct ratings compared to independent groups (Fig. 2A, right). This happens even if the average difficulty across all individuals is below 0.5.

*H2: When it is not too difficult for ideologically aligned individuals to classify a message correctly (d*_*align*_ *< 0.5), then individuals in segregated groups (with information about previous rating choices) classify true messages more often correctly than individuals in independent groups (without information about previous rating choices).*

*H3: When it is difficult for ideologically aligned individuals to classify a message correctly (d*_*align*_ *> 0.5), then individuals in segregated groups (with information about previous rating choices) classify false messages less often correctly than individuals in independent groups (without information about previous rating choices).*

The center panel of Fig. 2B illustrates the evolution of the rating signal when a true message originates in an ideologically aligned cluster, showing how the fraction of correct decisions by an agent’s position in a sequence (averaged over 10,000 simulation runs) is strictly higher in the segregated scenario than in the independent scenario. This can be attributed to the positive feedback loop that is likely to occur when a true message with low aligned difficulty (*d*_*align*_) accumulates an increasingly accurate rating signal. The center plot of Fig. 2B also shows how the fraction of correct decisions among misaligned individuals decreases in *i*’s position. Because for true messages *d*_*mis*_ > *d*_*align*_, rating accuracy will decrease to some extent among misaligned individuals. Conversely, if a false message originates in an ideologically aligned cluster (Fig. 2B, right panel), high aligned difficulty will trigger a negative feedback loop and the message accumulates an increasingly incorrect rating signal. Ratings will also recover to some extent once misaligned agents rate the false message because *d*_*mis*_ < *d*_*align*_. Taken together, we expect the following dynamics:

*H4: When it is not too difficult for ideologically aligned individuals to classify a message correctly (d_align < 0.5), then in segregated groups (with information about previous rating choices) classification accuracy first gradually improves among aligned individuals (H4a) and then gradually deteriorates among misaligned individuals (H4b).*

*H5: When it is difficult for ideologically aligned individuals to classify a message correctly (d*_*align*_ *> 0.5), then in segregated groups (with information about previous rating choices) classification accuracy first gradually deteriorates among aligned individuals (H5a) and then gradually improves among misaligned individuals (H5b).*

## Results

We tested our hypotheses by letting 80 groups of 50 participants sequentially rate true and false informational messages in an online experiment (*N* = 4000 participants with a total of 80,000 decisions). An equal number of self-reported conservative or liberal subjects rated informational messages that clearly supported either of the two ideological viewpoints. In doing so, we ensured that participants had systematic cognitive biases in favor of or against certain messages. Subjects were recruited from Amazon Mechanical Turk and Prolific. We implemented the three conditions studied in the simulations: a segregated condition (20 groups starting with liberals and 20 groups starting with conservatives), an integrated condition (20 groups), and an independent condition (another 20 groups). Each rating group featured 25 liberal and 25 conservative subjects. In each group, only one subject was active at a time to ensure a continuous, sequential evolution of real-time ratings. Subjects were recruited in small batches according to the number of available slots in experimental groups. Each subject answered to the same set of 20 messages, totaling 1,000 rating decisions per group. See Fig. 1, “Methods”, and Supplementary Information for details. Unless indicated otherwise, test results are derived from two-sided randomization tests. We test hypotheses separately for liberal and for conservative messages as to ensure a homogenous message sample in each step of the analysis.

### Integrated groups

**Figure 3:**
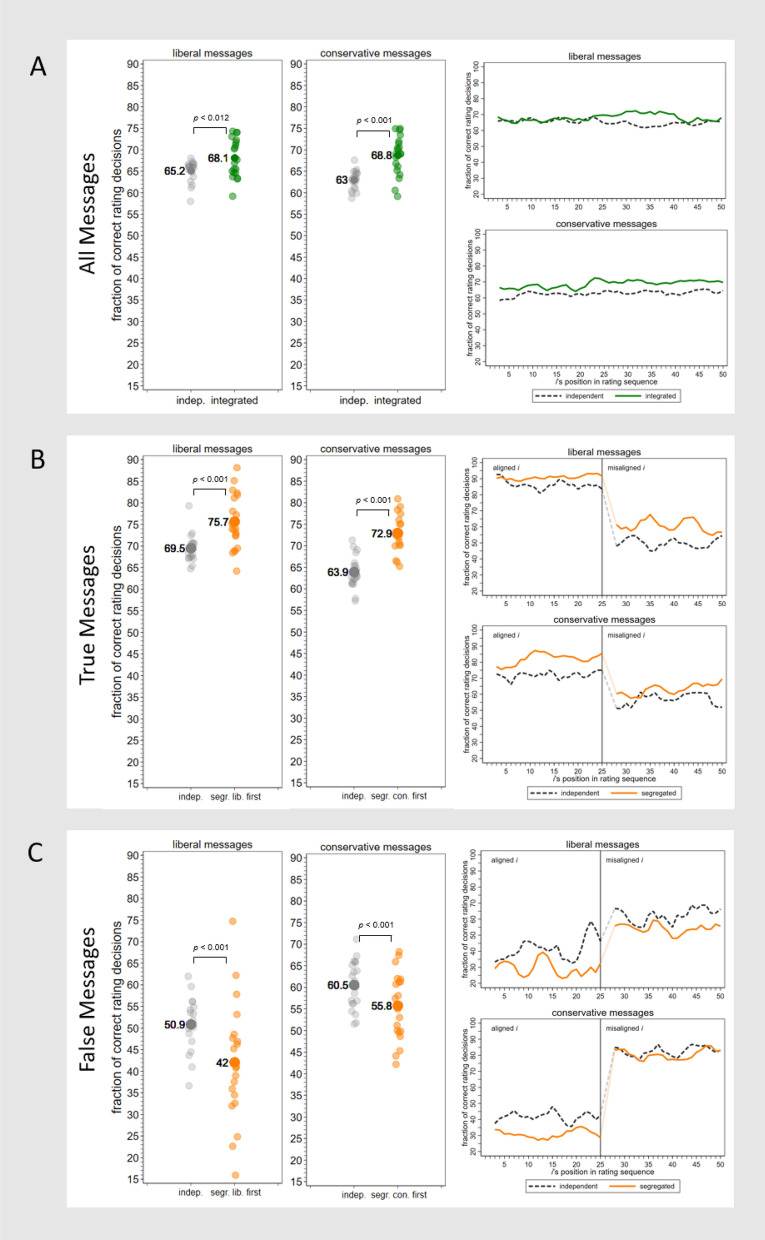
Broadcasting ratings enhances subjects’ rating accuracy in ideologically integrated groups (**A**) and, when messages are true, also in segregated groups (**B**). Ratings backfire in ideologically segregated groups when messages are false (**C**). Left side of the figure: Each small, shaded circle represents the number of correct rating decisions divided by all rating decisions in one group. Large circles represent means over groups. Right side of the figure: Lines represent moving averages over three subjects ($\overline{x }= \frac{{x}_{i}+ {x}_{i-1 }+ {x}_{i-2 }}{3}$. For this reason, data for subjects at the start of a sequence is not shown). *N*_*subjects*_ = 4000; *N*_*groups*_ = 80. All messages with $\overline{d }$ < 0.5; Panel B: true messages with *d*_*aligned*_ < 0.5. Panel C: false messages with *d*_*aligned*_ > 0.5. See “Methods”.

Consistent with Hypothesis 1, broadcasting the rating in ideologically integrated groups improved classification accuracy: The fraction of correct rating decisions in integrated groups was higher than in independent groups, both when liberal messages were rated (integrated 68.1% vs. independent 65.2%; *ATE* = 2.9%, *p* < 0.011, *N* = 40) and when conservative messages were rated (integrated 68.8% vs. independent 63.0%; *ATE* = 5.7%, *p* < 0.001, *N* = 40). Figure 3A illustrates this finding. Importantly, the fraction of correct rating decisions was higher irrespective of whether subjects rated true messages (integrated 71.6% vs. independent 66.7%; *ATE* = 4.9%, *p* < 0.001, *N* = 40) or false messages (integrated 65.0% vs. independent 61.1%; *ATE* = 3.9%, *p* = 0.049, *N* = 40). Aggregating over liberal and conservative messages further revealed a robust treatment effect, with 68.5% of rating decisions being accurate in integrated groups and 64.0% in independent groups (*ATE* = 4.4%, *p* < 0.001, *N* = 40).

### Segregated groups

Supporting Hypothesis 2, subjects were more often accurate if a true message was being rated and those who aligned with the connotation of the message were to do ratings first (Fig. 3B). In segregated groups where liberals rated liberal-leaning true messages first, the overall fraction of correct rating decisions rose by 6.2 percentage points as compared to independent groups rating the same messages (independent 69.5% versus liberal-first 75.7.%; *ATE* = 6.2%, *p* < 0.001, *N* = 40). Similarly, when conservatives rated conservative-leaning true messages first, the fraction of correct rating decisions increased by 9.0 percentage points (independent 72.9% versus conservative-first 62.9%; *ATE* = 9.0%, *p* < 0.001, *N* = 40).

The right side of Fig. 3B illustrates how broadcasting the rating in segregated sequences enhances rating performance: Compared to subjects in independent groups, the average propensity of a subject to make a correct rating decision in segregated groups increases after the first few ratings have been made, and then consistently stays above the average accuracy of subjects in independent groups. The large decreases in accuracy around the 25^th^ individual in Panel B reflect that aligned subjects are likely to rate aligned true messages as true while misaligned subjects are more likely to rate them as false.

Our third hypothesis postulates that ratings backfire when aligned subjects rate a *false* message first. Comparisons of independent and segregated groups in Fig. 3C show that this was indeed the case. In segregated groups where liberals rated liberal-leaning, false messages first, the fraction of correct rating decisions decreased from 50.1 percent in independent groups to 42.1 percent in liberal-first groups (*ATE* = 8.9%, *p* = 0.013, *N* = 40). In segregated groups where conservatives rated conservative-leaning, false messages first, the fraction of correct ratings sunk from 60.6 percent in independent groups to 55.7 percent in conservative-first groups (*ATE* = 4.8%, *p* = 0.033, *N* = 40). Backfiring becomes further visible in the right side of Fig. 3C: Real-time broadcasting of rating decisions decreases accuracy when aligned subjects make incorrect rating decisions in the beginning of a rating sequence, which influences subjects to make incorrect decisions later in the sequence.

Results of a multilevel logistic regression analysis show mixed evidence for Hypothesis 4 (see details in Supplementary Table S3). For true messages (H4a), aligned subjects’ likelihood to make a correct rating decision did increase relative to their position in a rating sequence among conservative subjects (*β* = 0.020, *p* = 0.02). However, this was not the case for liberal subjects (*β* = 0.015, *p* = 0.22). We did not find decreasing tendencies for misaligned subjects to make correct rating decisions when messages were true (H4b). The tendency for liberal misaligned subjects was positive (*β* = 0.019, *p* = 0.01) and not significant for conservative misaligned subjects (*β* = − 0.00, *p* = 0.44). Similarly, no evidence for Hypothesis 5 was found (details in Supplementary Table S4). Aligned subjects’ likelihood to make a correct rating decision did not decrease relative to their position in a rating sequence (H5a), both among liberal aligned subjects (*β* = − 0.007, *p* = 0.53) and among conservative aligned subjects (*β* = − 0.001, *p* = 0.87). Neither did we find increasing tendencies for misaligned subjects to make correct rating decisions (H5b), irrespective whether they were liberal (*β* = − 0.002, *p* = 0.89) or conservative (*β* = 0.004, *p* = 0.70). While patterns are much clearer on the macro-level, it is likely that individual idiosyncrasies contributed to the lack of clearly recognizable trends on the individual level: Our theoretical model assumed identical susceptibility to the rating signal and identical ability among subjects of the same ideological leaning to correctly classify information. In reality, subjects differ along those dimensions, contributing to noisier patterns that require more data for idiosyncrasies to cancel out. It is also thinkable that our participants acted more heuristically than the fine-grained interpretation of the rating signal used by agents in the simulation model. The rating signal may have only influenced subjects’ rating choices when the discrepancy between the count of true versus false rating decisions was sufficiently large. If this were the case, trends on the individual position level would become more static and less continuous than those suggested by the theoretical simulations in Fig. 2.

## Discussion

Our findings identify a key condition for the viability of real-time user ratings as an intervention against misinformation in online social networks: the presence of a sufficient degree of ideological mixing. In bipartisan environments with only moderate homophily, the enabling of real-time user ratings may succeed at tempering belief and reducing spread at the crucial early stage of propagation. Existing approaches such as professional fact-checking have not been able to intervene in such a timely manner. The availability of information on the veracity perceptions of previous others then allows individuals to more often correctly classify both true and false messages than in the absence of such information. While partisanship is often thought to amplify users’ belief in misinformation^2,41^, this finding speaks to the resilience of well-mixed, balanced bipartisan communities^45^. In ideologically segregated environments, by contrast, false information is more often incorrectly rated as true because systematically biased, early ratings mislead later decision-makers to make incorrect rating decisions as well. Such backfiring poses a challenge to the benefits of real-time ratings since many online social networks are marked by substantial ideological segregation^29,31–33^. Our study provides a systematic assessment of such backfiring effects in a controlled experimental setting.

In order to achieve this control, we had to build an artificial experimental environment that inevitably lacked contextual elements present on many real-world platforms. An example of such an element is the felt presence of like-minded others in segregated online spaces, which is known to shift individual behavior towards sharing information according to partisan identity rather than information veracity^14,30,32,46^. By hiding other community members’ ideological identities, our experiment excluded such corrupting effects on individual behavior. Another example is that we provided no incentivizes for correct veracity judgements. On the one hand that makes our environment match online social media that do not provide formal incentives either^47^, but on the other hand there may be reputational incentives on many platforms that lead some categories of users to care about veracity more on such platforms than in our experiment. Future research examining backfiring effects in more ecological environments would complement the present study, for example by investigating ideological segregation and the temporal evolution of veracity assessments on Twitter’s birdwatch or similar instruments on other platforms.

Given our study’s finding that real-time user ratings backfire in ideologically segregated environments, another important avenue for future research is therefore to explore how such backfiring can be prevented. One approach toward achieving this is the weighting of ratings by user ideology, which would prevent them from becoming inaccurate when users’ ideological biases are correlated with rating order or when populations are ideologically unbalanced. Alternatively, broadcasting a rating could be paused in highly homogenous environments until the rating is composed of a sufficiently diverse user base. Both these options, however, would require that the ideology of users be known or derived from earlier sharing and posting behavior. Moreover, initially paused broadcasting would come at the loss of potentially being able to warn users about false content early in the diffusion process.

The availability of a rating system can only limit the spread of misinformation if it influences sharing behavior. Our experiment only studied accuracy judgments, not the resulting sharing behavior. Another direction of future research is to investigate the consequences of rating systems for user behavior. One such consequence may be that users refrain from sharing information with a bad reputation because they do not want to risk misleading others^8^. Broadcasting ratings along with a message will also make visible who shares information that is likely false. This would make it easier for both network neighbors and the online platform to put users under scrutiny who repeatedly share information with a bad reputation. As a consequence, users might consider carefully if they want to share such information. Recent research suggests that positive social cues facilitate sharing of information more when it is true rather than false^48^. Future research may investigate if, conversely, users also avoid sharing information when social cues are negative, and whether this occurs out of fear of backlash, or out of intrinsic hesitation to spread potential falsehoods. Platforms may also consider incentivizing such reputational considerations to improve the functioning of user ratings: On Twitter’s birdwatch, users can only publish a rating if enough other users have identified their previous assessments as ‘helpful’. Reputational efforts can also be incentivized by exposing distinguished users to fewer advertisements, granting higher visibility to those with better reputations, or by rewarding especially diligent community members with ‘badge systems’ as already implemented on Facebook.

Crowd-based rating systems require that the rating signal is informative. Research suggests that online users are reasonably able to discern true content from false content most of the time^7,9,13,49^, and that they can be nudged to base their sharing decisions more on veracity, thus ‘de-biasing’ users’ assessments^13,50–52^. Of course, rating systems may be vulnerable to manipulation, e.g. by social bots or online trolls. This threat becomes particularly severe when malevolent behavior in one dominant ideological direction is concentrated among those who first rate a message, having similar adverse effects as the ratings of ideologically friendly users in segregated networks. These various limitations notwithstanding, we conclude that on ideologically integrated platforms, real-time user ratings can be a promising intervention for identifying misinformation early in its diffusion process and preventing users from believing in it. On highly segregated platforms, however, rating systems are likely to make things worse.

## Methods

The experiment was pre-registered on the Open Science Framework (https://osf.io/p5byq/) and approved by the Ethics Committee of the European University Institute, Florence. Data were collected between August 18 and December 31, 2021. All experiments and subsequent data handling were performed in accordance with relevant guidelines and regulations. We obtained informed consent from each participant prior to the experiment. The supplementary section ‘Recruitment of Experimental Subjects’ provides a detailed account of subject handling and consent procedures.

### Informational messages

We used 20 true and false informational messages with either a liberal or a conservative ideological connotation to be rated by the subjects. Subjects were instructed to ‘read 20 statements and click true if you believe a statement to be true and click false if you believe it to be false’. Within each experimental group, subject *i* had to complete rating all messages before subject *i*+1 could start their task. True messages summarized the main finding of a scientific article published in a social science or general science journal after 2015 as to provide a ground truth (example: “Gender diversity in student teams measurably improves their productivity”). False messages incorporated proven falsehoods by summarizing the inverted central finding of a published scientific article (example: “Human-induced CO2 levels in the air have no measurable impact on the likelihood of wildfires in California”). We ensured through pretesting that messages were indeed perceived as liberal or conservative leaning. Messages had the length of a tweet (< 280 characters) as to resemble pieces of information on online social media. We chose a balanced message set of 5 liberal and false, 5 liberal and true, 5 conservative and false, and 5 conservative and true messages. See supplementary section ‘Message Selection’ for a detailed account of the message set.

### Analytical strategy

We calculate average difficulty $\overline{d }$ (for each message) as the fraction of correct rating decisions by the total of all decisions in the independence condition. *d*_*align*_ is calculated as the fraction of correct rating decisions among those who align with the ideological connotation of a message by all rating decisions from this group. Since decisions are completely independent, values for $\overline{d }$ and *d*_*align*_ do not have to be computed at the sequence level but are aggregated over all decisions in that condition. Throughout the analyses, we only selected messages with $\overline{d }<0.5$. This was done to ensure that the average decision maker was better than random and to prevent a real-time rating from backfiring regardless of group composition. For each hypothesis, we then selected those messages that fell into the respective scope of the hypothesis, determined by the values of $\overline{d }$ and *d*_*align*_ (e.g. for the test of H2 we select only messages with *d*_*align*_ < *0.5).* To test Hypotheses 1–3, we use a non-parametric permutation test with 100,000 permutations. Hypothesis 4 and 5, unlike the other hypotheses, concerns individual rather than group behavior and thus requires an individual-level test. We use multilevel mixed-effects logistic regressions in which we regress individual rating decisions (correct vs. incorrect: 1/0) on the subjects’ position in the sequence (see Supplementary Table S3 and Supplementary Table S4). Note that $\overline{d }$ and *d*_*align*_ are estimates rather than ‘true values’. In the supplementary section ‘Robustness of Findings’, we present analyses taking statistical uncertainty of $\overline{d }$ and *d*_*align*_ into account and obtain similar results for H1, H2, H4 and H5 at conventional significance levels.

## Supplementary Information

Supplementary Information.

## Data Availability

Preregistration, all data and code available at https://osf.io/p5byq/.
