# Supplementary material for: Realtime user ratings as a strategy for combatting misinformation: an experimental study
Source: Sci Rep. 2023 Jan 28;13:1626. doi: 10.1038/s41598-023-28597-x (PMC9884269; doi:10.1038/s41598-023-28597-x)
Supplement: Supplementary file 1 — Supplementary Information. [file 41598_2023_28597_MOESM1_ESM.pdf]

Realtime user ratings as a strategy for combatting misinformation: An experimental study

Supplementary Information

Scientific Reports, 2023
DOI 10.1038/s41598-023-28597-x
Jonas Stein, Vincenz Frey, Arnout van de Rijt

Recruitment of Experimental Subjects

Measuring performance at the level of 50-subject groups, our study required many participants. We therefore recruited from two online crowd-working platforms, Amazon Mechanical Turk (MTurk) and Prolific. 40 of our 80 rating groups were occupied by subjects recruited from MTurk; the other 40 groups were recruited from Prolific. Each set of 40 groups comprised 10 groups for each type of group / treatment condition. Hence, both datasets comprised 2,000 subjects (1,000 liberals and 1,000 conservatives). On MTurk, data were collected between August 18 and October 12, 2021 and between November 10 and December 31, 2021 on Prolific. Participation was restricted to US residents to ensure a homogenous participant pool responsive to our informational messages. On MTurk, we recruited subjects by posting a general advertisement for US 'workers'. Prolific allows for a more selective targeting of participants, and for this reason, we posted separate advertisements for liberals or conservatives. Because a large share of US Prolific workers identifies as liberal or conservative but does not indicate this in the platform's pre-screening information, we also recruited Democrats and Republicans (approximately 20% of the Prolific dataset), and Biden versus Trump voters from the 2020 election (another 20% of the dataset). On Prolific, we excluded active MTurkers to ensure independence of observations. Apart from these slightly different recruiting procedures, the studies were identical. Because only one subject per group could be active at a time, recruitment of subjects was sequential, and advertisements were continuously adapted according to the availability of liberal or conservative slots in our experimental groups.

After having clicked on our advertisement on MTurk or Prolific, subjects were routed to a screener study in which they were asked a standard question about ideological self-identification[1]. Subjects identifying as moderate were remunerated $0.15 and excluded from the study. Remaining subjects were instructed on their task, indicated their informed consent, and were asked the ideological self-identification question a second time. Subjects whose ideological leaning did not match their self-reported ideology in the initial screener study were remunerated $0.15 and excluded. The self-identification question, informed consent form, and experimental instructions are presented in Supplementary Fig. S1.

[1] As implemented in the 2020 American National Election Studies Time Series Study: https://electionstudies.org/data-center/2020-time-series-study/

Subjects who completed their task successfully earned $1.5. When subjects entered the experiment, we informed them that they were to do ratings in ‘groups’ and, depending on the condition, explicated whether they could see others’ ratings or not. To mimic an online social media platform, where no financial incentives for a certain individual behavior are present, we chose to pay subjects flat fees instead of paying them for the accuracy with which they classified true and false messages.

Supplementary Figure S1. General Instructions.

Task & payment

Thank you for accepting this HIT. This academic research study should not take more than 8 minutes.

1. You will see 23 short informational statements, which are either true or false.
2. Your task is to read the statements and then **click ‘true’ if you believe a statement to be true or click ‘false’ if you believe it to be false**.

Example statement:

"Black and Hispanic students admitted to elite US colleges perform more poorly than Asian students." - True or false?

When you finish the HIT, you will receive a completion code in order to get paid. You will receive **$0.15 + $1.5** upon completion of the study.

Disclaimer

There are statements to check that you are **paying attention** and are not a robot. If you answer these statements incorrectly, if you **make your decisions too fast**, or if you **fail to finish** your task in **10 minutes**, you are excluded from this study. You can participate only once. **Please do not close this tab or reload the page during the task.** If you leave the website during the task, you will not receive any earnings.

Terms and Agreements

The data collected in this study does not include any personally identifying information about you. By participating, you understand that the research data gathered during this study will be used by the researchers. A dataset that contains your fully anonymous data may be published. A record of your workerID will be deleted after this study.

The data for this study is collected and controlled by Arnout van de Rijt of the European University Institute (EUI) and processed by Jonas Stein of the University of Groningen. Your data is protected by EUIs data protection policy (PD10/2019). You may contact EUI’s data protection officer through data_protection_officer@EUI.eu. You have the right to withdraw your consent for participating in this study at any time by closing this tab during the task. Upon withdrawal, your data will be deleted.

If you have questions concerning this study, please write to sociology.vanderijt@gmail.com

A copy of the consent form co-signed by the researcher can be obtained here.

Personal data

I have received sufficient information about this study and understand my role in it. The future processing of my personal data has been explained to me and is clear.

Terms of service

I have carefully read and understood the above information, agree to the terms for participation in this study, and am at least 18 years of age.

Before we start

Here is a 7-point scale on which the political views that people might hold are arranged from extremely liberal to extremely conservative. Where would you place yourself on this scale?

- extremely liberal
- liberal
- slightly liberal
- moderate, middle of the road
- slightly conservative
- conservative
- extremely conservative

Data Quality

Apart from checking for consistent ideological leaning, we undertook further measures to ensure high data quality. First, we excluded subjects who did not read messages carefully. We excluded subjects from further participation if they made a rating decision after having seen a message for less than three seconds thrice. Second, if a subject took more than 10 minutes to finish their task, they were excluded from the study. Third, we excluded subjects that failed at least one of our three attention check messages (example: "Europe is in the southern hemisphere." True / False). Subjects who failed one of these quality checks were only paid the remuneration for our screener study, $0.15. Excluded subjects were replaced with new subjects and their rating choices were not considered in the counts that were displayed as rating signals to subsequent subjects.

Of the 9,512 subjects who reacted on our study advertisement and went through the screener tasks, 3,284 (34.5%) were excluded because they identified as moderates, reported inconsistent ideological leaning, or had an active MTurk account parallel to their participation on Prolific. 684 (7.1%) subjects chose to not proceed with the experiment after the pre-screener or refused consent. 568 subjects (5.37%) were unable to participate because they showed up at a time when no spots for subjects of their ideological leaning were available. 975 subjects (10.3%) failed at least one of our quality checks: 511 subjects (5.4%) had unreasonably short response times, 387 (4.1%) failed an attention check question and 77 subjects (0.8%) did not finish within 10 minutes. Overall, participation rates were similar across samples. On MTurk, 41.4% of all individuals who reacted to our study advertisement (2,000 out of 4,834) successfully finished the study; on Prolific, it was 42.8% (2,000 out of 4,678).

Message Selection

Prior to the experiment, messages were calibrated through pretesting: Independent evaluations of 350 conservative and 350 liberal subjects ensured that liberal messages were more likely to be perceived as true by liberal subjects, and conservative messages more likely to be perceived as true by conservative subjects. Second, we ensured during the pretest phase that subjects were more likely to make a correct rather than an incorrect evaluation of a messages' veracity: The median rating of a message always had to reflect the actual veracity of the message. Put differently, each message had to have an average difficulty below 0.5 and a bias greater zero. Both are scope conditions of this study: The wisdom of crowds requires that more than 50 percent of the population make a correct rating decision in independence [18,20], and there must be a difference in message difficulty among aligned versus misaligned subjects for a segregated rating orders to have any effect at all. Out of an original set of 144 messages used for pretesting, we chose a subset of 20 messages as compared to more messages to prevent subjects getting tired or inattentive after too many messages. Supplementary Table S1 presents an overview of the message set used in the experiment.

Supplementary Table S1. Message set in the experiment.

ID	true	lib.	text	d_{align}	$\bar{d}$	Hypoth.
1	0	0	Human-induced CO2 levels in the air have no measurable impact on the likelihood of wildfires in California	.48	.32	1
2	0	0	Black people have similar chances of receiving a job offer than white people, as long as they are similarly qualified	.70	.43	1, 3, 4
3	0	0	Children raised by homosexual parents are 10 percent more likely to experience mental health issues than children raised by heterosexual parents	.51	.32	1, 3, 4
4	0	0	Affirmative action has reduced the number of highly qualified applicants for specialized jobs by roughly 40 percent	.56	.37	1, 3, 4
5	0	0	US states enacting major tax cuts for the wealthy show higher growth rates and economic prosperity, also for poor people, than states without tax cuts	.48	.45	1, 3, 4
6	1	0	Cannabis use is associated with lasting damage to adolescents' cognitive functions	.36	.40	1, 2
7	1	0	Most gun control restrictions generally have had little effect on violent crime in US cities	.17	.37	1, 2
8	1	0	Children born to married parents have slightly better health at age 5 than children born to unmarried parents	.28	.32	1, 2
9	1	0	Unmarried couples are more likely to have additional sexual partners as compared to married couples	.27	.38	1, 2
10	1	0	The United States have won more medals at the Summer Olympics than any other nation	.30	.33	1, 2
11	0	1	Large influxes of migrants strongly increase the number of jobs available to native applicants of the host country	.37	.33	1
12	0	1	In countries run by left-wing political parties, immigrants integrate and learn the spoken language of the host country nine times faster than in right-wing countries	.67	.50	-
13	0	1	Men in same sex relationships are more likely to be in serious relationships than heterosexual men	.33	.30	1
14	0	1	Due to increasing inequalities in US healthcare provision, cancer patients in the US have 85 percent lower survival chances than patients in both eastern and western Europe	.61	.49	1, 3, 4
15	0	1	Israel's construction of a southern border wall between 2010 and 2013 was not effective, as the annual numbers of illegal crossings increased by 80 percent	.58	.49	1, 3, 4
16	1	1	Due to safe jobs and fixed wages, eastern Europeans felt economically more secure under socialism than under capitalism	.30	.41	1, 2
17	1	1	Police officers speak significantly less respectfully to black community members than to white ones in everyday traffic stops	.22	.33	1, 2
18	1	1	Gender diversity in student teams measurably improves their productivity	.10	.25	1, 2
19	1	1	When Latino immigrants move into an area, there is no measurable increase in homicide rates	.12	.26	1, 2
20	1	1	Germany's relative income equality has resulted in longer life expectancies for Germans as compared to Americans	.12	.28	1, 2

Subjects' Rating Behavior

Consistent with the scope conditions of this study, subjects were reasonably able to tell true from false messages in the independent sequences of the experiment. On average, subjects in the independent condition thought 66.7% of true messages to be true, while this was the case for only 40.1% of false messages (paired t-test: $t = 34.5$, $p < 0.001$, $N = 500$). At the same time, subjects in the independent condition found 66.2% of messages that aligned with their ideology to be true, but only 40.5% of misaligned messages (paired t-test $t = 28.0$, $p < 0.001$, $N = 500$). This shows that indeed, cognitive biases played a role in such a manner that messages supporting one's own viewpoint are more often thought to be true – independent of the actual veracity of a message. It is noteworthy that liberals were especially inclined to find liberal true messages true (85.7% of messages in this category), while this was rarely the case for false conservative messages (19.1%). Classifying false conservative messages as false and liberal true messages as true made liberals better at making correct rating decisions overall (average liberal subject: 67% correct vs. conservative subject: 59%; t-test $t = 11.3$, $p < 0.001$, $N = 500$). At the same time, liberal subjects were more biased than conservatives: The difference between finding aligned versus misaligned messages true was larger among liberals (30.1 pp.) as compared to conservatives (20.1 pp.; t-test $t = 5.5$, $p < 0.001$, $N = 500$). An overview of subject behavior by message veracity and message ideology is presented in Supplementary Table S2. Overall, independent subjects rated 63.5% of their messages correctly, suggesting that ability in the population was indeed above 0.5 for at least a sizeable portion of all messages. Subjects in the independent condition of the Prolific sample were slightly better at making correct rating decisions than in the MTurk sample (64.8% versus 61.9%, t-test $t = 3.8$, $p < 0.001$, $N = 500$). Simultaneously, the difference in finding aligned versus misaligned messages to be true (bias) was higher among Prolific subjects as compared to MTurk subjects (Prolific: 31.5 pp., MTurk: 19.9 pp.; t-test $t = 6.4$, $p < 0.001$, $N = 500$). Both differences are consistent with prior studies finding subject quality higher on Prolific[51,52] – subjects may have read questions more closely and thought of them more proactively. Indeed, a much lower number of quality check failures (94 subjects on Prolific versus 881 subjects on MTurk) suggests that fewer subjects showed satisficing behavior on Prolific. Because subject behavior was slightly different in the Prolific dataset as compared to the MTurk dataset, the Supplementary Robustness of Findings section reports additional analyses where underlying message parameters were constructed for each dataset separately.

Supplementary Table S2. Subject behavior in the independence condition

Subject ideology	Message ideology	Message veracity	% thought true[1]
conservative	conservative	false	56.6
		true	72.4
	liberal	false	33.2
		true	54.3
liberal	conservative	false	19.1
		true	55.5
	liberal	false	51.3
		true	84.5

[1] per cell: $N_{subjects}$ = 500; $N_{decisions}$ = 2,500

Supplementary Table S3. Regression results Hypothesis 4

Multilevel logit regression on making a correct rating decision

	Segregated (Liberal first)		Segregated (Conservative first)	
	Coef.	SE	Coef.	SE
aligned subject				
constant	2.520 ***	0.21	1.430 ***	0.14
$position_i$ (1-25)	0.015	0.01	0.020 *	0.01
misaligned subject				
constant	-0.103	0.29	0.880 *	0.41
$position_i$ (26-50)	0.019 *	0.01	-0.008	0.01
N Observations	1.000		2.000	
N Subjects	500		500	
N Sequences	20		20	

The regression models include an individual-level and a sequence-level variance term in the regression, and standard errors are clustered at the individual level. Only decisions for true messages with $d_{align} < 0.5$; $\bar{d} < 0.5$. * $p < 0.05$; ** $p < 0.01$; *** $p < 0.001$ (two-sided)

Supplementary Table S4. Regression results Hypothesis 5

Multilevel logit regression on making a correct rating decision

	Segregated (Liberal first)		Segregated (Conservative first)	
	Coef.	SE	Coef.	SE
aligned subject				
constant	-0.988 ***	0.25	-0.914 ***	0.17
$position_i$ (1-25)	-0.007	0.01	-0.001	0.01
misaligned subject				
constant	0.299	0.48	1.563 ***	0.42
$position_i$ (26-50)	-0.002	0.01	0.004	0.01
N Observations	1.000		2.000	
N Subjects	500		500	
N Sequences	20		20	

The regression models include an individual-level and a sequence-level variance term in the regression, and standard errors are clustered at the individual level. Only decisions for false messages with $d_{align} > 0.5$; $\bar{d} < 0.5$. * $p < 0.05$; ** $p < 0.01$; *** $p < 0.001$ (two-sided)

Robustness of Findings

We report two analyses of the robustness of our findings. First, since subjects from the MTurk dataset showed slightly different behavior than subjects from the Prolific dataset (see section S4), we treat subjects from each dataset as different populations and compute message parameters for each dataset separately. Second, we take into account that message parameters are estimates and not true values and only included messages whose 95 % confidence interval of average difficulty $\bar{d}$ did not overlap with the 0.5 threshold. For Hypotheses 2, we excluded all messages for which the upper confidence bound of d_{align} was below 0.5 and for Hypotheses 3 and 4, we excluded all messages for which the lower confidence bound was above 0.5. Note that since parameters were computed separately per dataset, sample sizes were smaller and standard errors larger. Supplementary Table S4 presents an overview of how many messages were selected for each Hypothesis and dataset. We then computed the fraction of correct rating decisions per sequence and conducted the same analyses as in the main results section.

Supplementary Table S5. Number of selected messages per hypothesis

Hypothesis	Mturk	Prolific
1	16	18
2	10	10
3	2	3
4	2	3
Messages	20	20

Consistent with Hypothesis 1, broadcasting ratings in integrated sequences led to an improvement in rating performance. In integrated groups, the fraction of correct rating decisions was higher than in independent sequences, both among liberal messages (72.7% versus 68.5%; *ATE* = 4.2%, $p < 0.001$, N = 40) and conservative messages (69.3% versus 63.7%; *ATE* = 5.6%, $p < 0.001$, N = 40). The additional results also support Hypothesis 2. Subjects were better at making correct rating decisions in groups where those who aligned with the connotation of a message were to do ratings first. In segregated groups where liberals rated first, the fraction of correct rating decisions rose by 5.7 percentage points as compared to independent groups (independent 71.4% versus liberal-first 77.0.%; two-sided randomization test: *ATE* = 5.7%, $p < 0.001$, N = 40). In segregated groups where conservatives rated first, average accuracy increased by 9.0 percentage points (independent 63.9% versus conservative-first 72.9%; two-sided randomization test: *ATE* = 9.0%, $p < 0.001$, N = 40).

Unlike the results in the main text, the fraction of correct ratings did not decrease significantly in segregated groups when d_{align} was above 0.5. However, results indicated effects of identical direction and similar strength: In segregated groups where liberals rated first, the fraction of correct ratings decreased by 6.6 percentage points (independent 55.2% versus liberal-first 48.6.%; two-sided randomization test: *ATE* = 6.6%, $p = 0.126$, N = 20). In segregated groups where conservatives rated first, the fraction of correct ratings sunk by 3.4 percentage points as compared to independent sequences (independent 54.4% versus conservative-first 57.8.%; two-sided randomization test: *ATE* = 3.4%, $p = 0.108$, N = 40).

Consistent with the main results for Hypothesis 4, a multilevel logit regression for true messages did show significant increasing rating performance over conservative aligned individuals' positions ($\beta = .019$, $p = .01$), but no increasing performance for liberal aligned subjects. No decreasing performance among misaligned conservative or liberal subjects was found. Finally, in line with the main results for Hypothesis 5, no significant decreasing rating performance over aligned individuals' positions in rating groups was present; or increasing performance for misaligned subjects. We conclude that results from our robustness analyses are similar to those in the main text – Hypotheses 1, 2 and 4 allowed for identical conclusions, and Hypothesis 3 achieved similar effects despite lack of significance. The lack of significance for H3 can likely be attributed to the fact that fewer messages were considered in the robustness analysis, and hence that less statistical power was given.
